# Personalizing medicine in Africa: current state, progress and challenges

**DOI:** 10.3389/fgene.2023.1233338

**Published:** 2023-09-19

**Authors:** Paul Owolabi, Yagoub Adam, Ezekiel Adebiyi

**Affiliations:** ^1^ Covenant Applied Informatics and Communication, Africa Centre of Excellence (CApIC-ACE), Covenant University, Ota, Ogun State, Nigeria; ^2^ Department of Computer and Information Science, Covenant University, Ota, Ogun State, Nigeria; ^3^ Covenant University Bioinformatics Research (CUBRe), Covenant University, Ota, Ogun State, Nigeria; ^4^ Applied Bioinformatics Division, German Cancer Research Center (DKFZ), Heidelberg, Germany

**Keywords:** personalized medicine, individualized medicine, precision medicine, genomics, Africa

## Abstract

Personalized medicine has been identified as a powerful tool for addressing the myriad of health issues facing different health systems globally. Although recent studies have expanded our understanding of how different factors such as genetics and the environment play significant roles in affecting the health of individuals, there are still several other issues affecting their translation into personalizing health interventions globally. Since African populations have demonstrated huge genetic diversity, there is a significant need to apply the concepts of personalized medicine to overcome various African-specific health challenges. Thus, we review the current state, progress, and challenges facing the adoption of personalized medicine in Africa with a view to providing insights to critical stakeholders on the right approach to deploy.

## 1 Introduction

**FIGURE 1:**
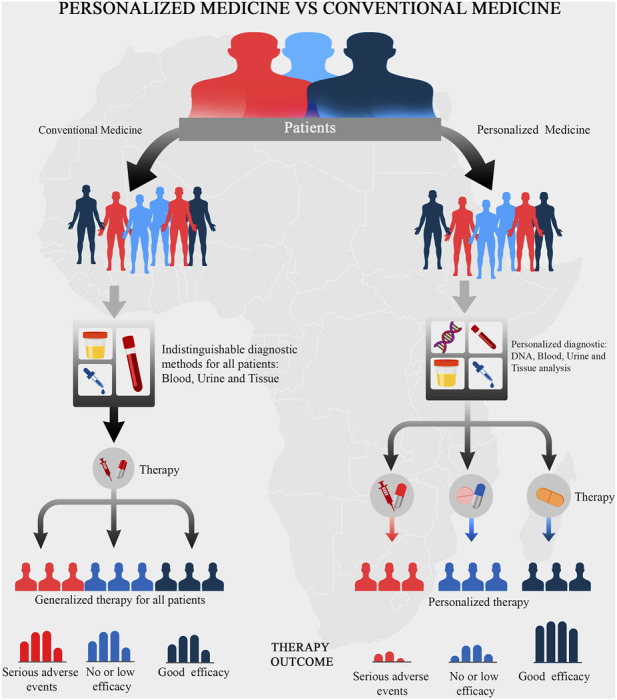
A schematic representation of the concept underlying personalized medicine. The underlying concepts of personalized medicine aims to prioritize the outcome of the medical treatment.

All over the world today, there is focused attention on personalized medicine as a way of revolutionizing healthcare delivery. Personalized medicine is a revolutionary form of medicine that utilizes an individual’s genetic, proteomic, and environmental information in the prevention, diagnosis, monitoring and treatment of diseases (McGonigle, 2016). It holds the potential of providing tailor-made medical interventions which maximize health benefits and minimize treatments’ side effects (McGonigle, 2016). This approach to preventing and/or treating diseases is hinged on taking each individual’s peculiarities into account (Collins and Varmus, 2015). The scientific basis underlying the concept of personalized medicine is that individual-level genomic information can be investigated and used significantly in clinical usage as well as in providing strategies for public health policies, See Figure 1. The concept underlying individualized healthcare has become a source of great hope to treat many complex diseases, including cancer (Ghoorah et al., 2021; Hasanzad et al., 2021). This concept might help Africans in overcoming many diseases that are related to the African continent. For example, studies have revealed that Africans have a seemingly high risk of developing chronic kidney disease (CKD) at an early age and with a faster progression toward kidney failure (George et al., 2021). Thus, personalized medicine strategies may be adopted to mitigate this and several other health conditions.

Personalized medicine entails optimizing drug choice, dosage, and treatment regimen while avoiding adverse drug effects for the specific patient. Its goal is to make patient-specific prescriptions of medicine and treatment based on their genetic makeup. Genetic variants could potentially play critical roles in the efficient administration of a particular drug, i.e., absorption, distribution, metabolism, and excretion of active components (ADME) (Adeyemo and Rotimi, 2014; Sigman, 2018). However, the application of personalized medicine might be limited when applied to non-genetic diseases. For instance, the burden of non-communicable diseases is largely considered to be as a result of lifestyle changes and urbanization (Collins and Varmus, 2015; Drake et al., 2018; Afolaranmi et al., 2021) rather than individualized genetic changes. Nonetheless, the implementation of personalized medicine will result in a paradigm shift from the conventional symptom-based, “trial-and-error” health system to a genome-based approach, thereby, bringing about precision and personalization in disease diagnosis, management, and treatment (Drake et al., 2018).

This review is aimed at highlighting the giant strides recorded so far in the journey to personalizing medicine in Africa as well as identifying some of the current challenges faced in its translation into routine clinical practice. It also seeks to proffer insights on how these challenges can be effectively tackled.

## 2 The current state of personalized medicine in Africa

**FIGURE 2:**
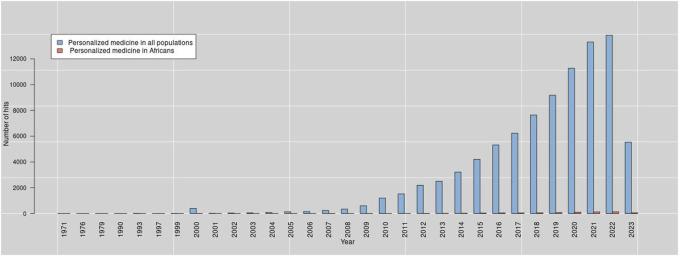
Progress of research in personalized medicine from the year 1971–2023.

The term “personalized medicine” was introduced to the scientific community in 1971 (Gibson, 1971). However, the advent of personalized medicine in Africa is still evolving. For instance, by querying PubMed for personalized medicine-related terms on 15 May 2023, we obtained a total of 79,273 hits. The histogram of these hits demonstrates that there is an exponential growth of the publications in this area, see Figure 2. We used the search syntax below for querying PubMed for personalized medicine:

((“personalized medicine”) OR (“individualized medicine”) OR (“precision medicine"))

However, to restrict the hits for personalized medicine in Africa, we used the search syntax below:

(((“personalized medicine”) OR (“individualized medicine”) OR (“precision medicine")) AND (“Africa"))

Although few authors have argued about the subtle distinction in the terms ‘personalized medicine’, ‘individualized medicine’, and ‘precision medicine’, we used them synonymously in our search query since most authors on the subject have used them interchangeably (Khoury, 2016; Goetz and Schork, 2018; Sugeir and Naylor, 2018). For more details about the above PubMed query syntax, refer to Bramer et al. (2018). We obtained only 620 hits (0.78%) when we restricted our results to Africa and surprisingly the scientific effort started after 30 years, i.e., started in 2000. Moreover, when we restricted our search to Clinical Trials or Randomized Controlled Trials, we obtained only 21 hits (1.17%) for Africa out of 1,791 hits. The outcome of these 21 hits is summarized in Table 1. It is worth noting that by looking at the authors’ list of these 21 articles, we observed that there are only 46 researchers from Africa affiliated with 34 African institutes. Thus, it is safe to say that many developing countries in Africa still lack the expertise and technologies necessary to adopt genomic medicine widely, yet they continue to face challenges caused by the growing number of noncommunicable diseases (like cancer, diabetes, heart disease, and other chronic illnesses), as well as infectious diseases that are emerging and re-emerging (Kaze et al., 2018; Mulder et al., 2018; Mapesi and Paris, 2019; Mudie et al., 2019). As a result of these factors, Africa’s healthcare system is heavily burdened (Schlebusch et al., 2012).

**TABLE 1:** Summary of the 21 PubMed hits on the Clinical Trials or Randomized Controlled Trials in Africa.

S/N	Title	Publication year	Disease treated/managed	Study description	% Affiliation to African institutions
1	“Production of high specific activity (195 m) Pt-cisplatinum at South African Nuclear Energy Corporation for Phase 0 clinical trials in healthy individual subjects” (Zeevaart et al., 2013)	2013	Cancer	This clinical pilot study produced and provided (195 m)Pt-cisplatinum for a Phase 0 trial on healthy subjects. The high specific activity of the product allowed for a huge drop in the chemical dose of carrier cisplatinum, resulting in a well-tolerated and promising imaging agent with potential applications in personalized medicine	100.0% (n = 1)
2	“Efficacy and safety of fluticasone furoate/vilanterol compared with fluticasone propionate/salmeterol combination in adult and adolescent patients with persistent asthma: a randomized trial” (Woodcock et al., 2013)	2013	Asthma	This study compared the potency of the combination of fluticasone furoate (FF) and vilanterol VI) with fluticasone propionate (FP) and salmeterol (SAL) in patients with uncontrolled persistent asthma. The once-daily FF/VI treatment showed similar potency in enhancing lung function in comparison to the twice-daily FP/SAL treatment, with no significant differences in safety or adverse events	12.5% (n = 8)
3	“Fluticasone furoate-vilanterol 100–25 mcg compared with fluticasone furoate 100 mcg in asthma: a randomized trial” (Bleecker et al., 2014)	2014	Asthma	This study compared the effectiveness and safety of combining fluticasone furoate (FF) and vilanterol VI) *versus* FF alone in patients with persistent asthma. Both treatments significantly improved lung function in comparison to placebo, with the combination therapy showing a slightly greater improvement, but with a small risk of cortisol suppression	11.1% (n = 9)
4	“Effect of changes in BMI and waist circumference on ambulatory blood pressure in obese children and adolescents” (Hvidt et al., 2014)	2014	Obesity	This study investigated the influence of changes in weight on ambulatory blood pressure (ABP) in severely obese children and adolescents undergoing lifestyle intervention. The results showed that reductions in obesity measures were strongly correlated with changes in ABP but not clinic blood pressure (BP) after 1 year, highlighting the importance of using 24-h ABP measurements in this population	33.3% (n = 3)
5	“Efficacy and safety of fluticasone furoate 100 μg once-daily in patients with persistent asthma: a 24-week placebo and active-controlled randomized trial” (Lötvall et al., 2014)	2014	Asthma	The study examined the effectiveness and safeness of fluticasone furoate (FF) taken once daily *versus* a placebo for 24 weeks in asthma patients. Results showed that the treatment largely improved lung function and reduced rescue inhaler use, similar to the standard treatment of fluticasone propionate (FP) taken twice daily. Both treatments were well-tolerated, with fewer severe asthma exacerbations in comparison to the placebo	11.1% (n = 9)
6	“Weight reduction and aortic stiffness in obese children and adolescents: a 1-year follow-up study” (Hvidt et al., 2015)	2015	Weight reduction and aortic stiffness	This study examined the effects of decrease in weight on aortic stiffness in obese children and adolescents. After a 1-year treatment, aortic stiffness increased, primarily due to age-related factors, and was not linked to changes in obesity. Changes in another measure of arterial function were found to be linked with changes in abdominal and overall body fat. The impact of weight reduction on this group’s aortic stiffness was unclear	25.0% (n = 4)
7	“Tiotropium or salmeterol as add-on therapy to inhaled corticosteroids for patients with moderate symptomatic asthma: two replicate, double-blind, placebo-controlled, parallel-group, active-comparator, randomized trials” (Kerstjens et al., 2015)	2015	Asthma	This study tested the safeness and efficacy of tiotropium as an additional treatment for patients with moderate symptomatic asthma. It was found out that both the 5 μg and 2.5 μg doses better helped lung function and asthma control unlike the placebo. Tiotropium was also well-tolerated and could be considered as an alternative to salmeterol for this group of patients	10.0% (n = 10)
8	“Extracellular matrix biomarker, fibulin-1 and its association with soluble uPAR in a bi-ethnic South African population: the SAfrEIC study” (du Plooy et al., 2015)	2015	Sclerotic disease	The study found that fibulin-1 levels were independently associated with soluble urokinase-type plasminogen activator receptor (suPAR) and albumin in African men and with suPAR in Caucasian men. These results suggest a potential link between subclinical inflammation, extracellular matrix changes, and the development of cardiac fibrosis or vascular sclerosis in South African men with lower albumin levels	50.0% (n = 4)
9	“Predicting the effects of blood pressure-lowering treatment on major cardiovascular events for individual patients with type 2 diabetes mellitus: results from Action in Diabetes and Vascular Disease: Preterax and Diamicron MR Controlled Evaluation” (van der Leeuw et al., 2015)	2015	Diabetes and Vascular Disease	The study analyzed the result of treatments that lower blood pressure in type 2 diabetes mellitus patients and identified a multivariable treatment algorithm to determine subjects who benefit the most. The algorithm showed that risk reduction was predicted in 43% of patients, highlighting the potential for personalized treatment decisions in diabetes management	12.5% (n = 8)
10	“Blood pressure variability predicts cardiovascular events independently of traditional cardiovascular risk factors and target organ damage: a LIFE substudy” (Vishram et al., 2015)	2015	Cardiovascular disease	According to this study, high variability in blood pressure measurements during treatment may be linked to an aggravated likelihood of cardiovascular events and stroke in hypertensive individuals with left ventricular hypertrophy (LVH). However, it was not associated with heart damage or heart attacks. Monitoring blood pressure variability could be important for assessing the effectiveness of antihypertensive treatment in these patients	8.3% (n = 12)
11	“Increased hsCRP is associated with higher risk of aortic valve replacement in patients with aortic stenosis” (Blyme et al., 2016)	2016	Aortic stenosis	This study investigated the relationship between inflammation and aortic valve stenosis (AS) using the measurement of high-sensitivity C-reactive protein (hsCRP) levels at baseline and after 1 year. Elevated hsCRP levels at 1 year and an increase during the first year were associated with a higher likelihood of requiring aortic valve replacement (AVR), independent of other risk factors	14.3% (n = 7)
12	“Physiological Phenotyping for Personalized Therapy of Uncontrolled Hypertension in Africa” (Akintunde et al., 2017)	2017	Hypertension	This study conducted in Africa found that personalized treatment on the basis of renin/aldosterone phenotyping massively improved blood pressure control in African hypertensive patients who were not responding well to standard treatment. This approach shows promise for improving blood pressure management in Africans and African Americans with resistant hypertension	60.0% (n = 5)
13	“Genotype scores predict drug efficacy in subtypes of female sexual interest/arousal disorder: A double-blind, randomized, placebo-controlled cross-over trial” (Tuiten, Michiels, et al., 2018)	2018	Female sexual interest/arousal disorder	In this research, a genotyping-based procedure was used in predicting drug response in female sexual interest/arousal disorder. This personalized medicine approach shows promise in treating female sexual interest/arousal disorder	12.5% (n = 16)
14	“Efficacy and Safety of On-Demand Use of 2 Treatments Designed for Different Etiologies of Female Sexual Interest/Arousal Disorder: 3 Randomized Clinical Trials” (Tuiten, van Rooij, et al., 2018)	2018	Female sexual interest/arousal disorder	The study explored on-demand treatments for female sexual interest/arousal disorder (FSIAD) using personalized medicine. Testosterone combined with sildenafil improved sexual events in women with low sensitivity, while testosterone with buspirone increased sexual events in women with overactive inhibition. These treatments hold the potential for managing FSIAD.	11.1% (n = 18)
15	“Genome-wide association study of primary open-angle glaucoma in continental and admixed African populations” (Bonnemaijer et al., 2018)	2018	Glaucoma	This GWAS study in African populations revealed a strong association between primary open-angle glaucoma (POAG) and a genetic risk score that combines the effects of previously reported POAG loci. A novel candidate locus, rs141186647, was also identified, but validation was hampered by genetic heterogeneity	20.0% (n = 30)
16	“An omics-based strategy using coenzyme Q10 in patients with Parkinson’s disease: concept evaluation in a double-blind randomized placebo-controlled parallel group trial” (Prasuhn et al., 2019)	2019	Parkinson’s disease	This study was designed to evaluate potential benefits of coenzyme Q10 treatment in genetically stratified subgroups of Parkinson’s disease patients with an enrichment of risk variants in mitochondrial genes. Its findings hold the possibility of influencing personalized patient care and enhancing understanding of brain energy metabolism in Parkinson’s disease	10.0% (n = 10)
17	“Randomized Trial of an Intervention to Improve Blood Pressure Control in Stroke Survivors” (Owolabi et al., 2019)	2019	Stroke	The study tested whether a THRIVES (Tailored Hospital-based Risk reduction to Impede Vascular Events after Stroke) intervention which included personalized text messages, educational videos, and a patient report card could improve blood pressure control in stroke patients. Although the intervention did not significantly reduce blood pressure overall, there was a significant decrease in a subgroup of patients with baseline hypertension, suggesting that further research is needed to determine if text messaging and financial incentives alone can improve blood pressure control in stroke survivors	70.0% (n = 10)
18	“Cancer prevention with aspirin in hereditary colorectal cancer (Lynch syndrome), 10-year follow-up and registry-based 20-year data in the CAPP2 study: a double-blind, randomized, placebo-controlled trial” (Burn et al., 2020)	2020	Cancer	This 10-year study assessed the long-term effects of regular aspirin intake in individuals with Lynch syndrome and found it to greatly decrease the risk of colorectal cancer. It included subjects recruited from different parts of the world including Africa	5.3% (n = 19)
19	“Pharmacogenetic predictors of nevirapine pharmacokinetics in Ghanaian children living with HIV with or without TB coinfection” (Langaee et al., 2021)	2021	HIV	Researchers studied how genetic variations in drug disposition genes affect the way nevirapine (NVP) is processed in Ghanaian children with HIV. They discovered that specific genetic variations in CYP2B6 and NR1I2 genes could predict NVP’s behavior in the body. Identifying these genetic markers could help tailor NVP dosing for better treatment outcomes in HIV-infected children	33.3% (n = 9)
20	“Detection of HIV-1 Transmission Clusters from Dried Blood Spots within a Universal Test-and-Treat Trial in East Africa” (Pujol-Hodge et al., 2022)	2022	HIV	This trial aimed to reduce regional HIV-1 incidence through universal test-and-treat (UTT) in rural Uganda and Kenya. The phylodynamic analysis identified within-community transmissions and the presence of transmitted drug resistance, emphasizing the importance of improving ART delivery and adherence to prevent HIV-1 transmission in UTT settings	36.4% (n = 11)
21	“Cancer Prevention with Resistant Starch in Lynch Syndrome Patients in the CAPP2-Randomized Placebo-Controlled Trial: Planned 10-Year Follow-up” (Mathers et al., 2022)	2022	Lynch Syndrome (Cancer)	In this extensive CAPP2 trial, it was found that 30 g daily intake of resistant starch (RS) daily lowered the occurrence of non-colorectal cancers in individuals with Lynch syndrome (LS) over a 20-year period. Although there was no notable impact on colorectal cancer rates, RS showed a protective effect against non-colorectal LS cancers, especially those affecting the upper gastrointestinal tract	5.9% (n = 17)

● Total African-affiliated researchers = 46; total number of institutes = 34.

A simple breakdown of the results of the PubMed search shows that most of the hits were studies on conditions such as asthma, cancer, HIV, female sexual interest/arousal disorders, obesity, and cardiovascular diseases among others. The results obtained from these studies are summarized as follows.

A total of four studies have been done to investigate the application of personalized medicine in Asthma. The underlying samples were grouped based on disease status, i.e., persistent asthma *versus* intermittent asthma, or based on the patient’s reaction to the treatment. In detail, Woodcock et al. (2013) performed a randomized, double blind, double dummy, parallel group trial design. Their study involved 806 patients to assess the potency of fluticasone furoate-vilanterol combination (FF/VI) in comparison with fluticasone propionate (FP)/salmeterol (SAL) in asthmatic patients. The study concluded that the once-daily FF/VI was capable of improving lung function in the group of patients with persistent asthma. In 2014, Bleecker et al. (2014) investigated the efficacy and safety of the combination of corticosteroid fluticasone furoate and vilanterol (FF-VI) in comparison with fluticasone furoate (FF) in 609 asthmatic patients. They observed that FF-VI and FF resulted in improved lung function than placebo in the group of patients with persistent asthma. Furthermore, Lötvall et al. (2014) assessed the effectiveness and safety of the novel inhaled corticosteroids (ICS) fluticasone furoate (FF) compared to placebo. They reported that treating patients older than 12 years with FF 100 μg or 200 μg once daily will improve lung function in the group of patients with moderate-severe persistent asthma. In 2015, Kerstjens et al. (2015) investigated the safety and effectiveness of tiotropium in moderately asthmatic patients who showed symptoms despite being treated with medium-dose of ICS. They concluded that a once-daily tiotropium add-on to medium-dose inhaled corticosteroids lowers airflow obstruction and improves asthma control in the group of patients with moderate symptoms. Also, they considered Tiotropium to be a safe and potent bronchodilator and a possible substitute for salmeterol in this patient population.

Regarding cancer diseases, three studies investigated the utility of personalized medicine based on the underlying ethnic groups of the participants, i.e., participants are grouped based on their ancestry information. In detail, Zeevaart et al. (2013) performed a clinical trial for evaluating a cisplatinum product on 10 healthy volunteers. Although they used a relatively small sample size; however, they considered this product to be a promising imaging agent with potential applications in personalized medicine for the group of patients who are subject to platinum chemotherapy. In 2020, Burn et al. (2020) investigated Lynch syndrome which is linked with an increased risk of colorectal cancer, as well as with many different cancers (Burn et al., 2020). They conducted a double-blind, randomized CAPP2 trial. The total number of participants was 861 patients from 43 distinct international research centers worldwide. The participants were grouped based on their underlying ancestry as follows: 707 Europeans, 112 Australians, 38 Africans, and 4 Americans. The patients were randomly assigned to receive 600 mg aspirin (n = 427) or placebo (n = 434). The researchers monitored cancer outcomes up to 10 years from recruitment. The study’s findings reported that aspirin could be considered as a good preventive agent for colorectal cancer in group of pateints with Lynch syndrome. In 2022, Mathers et al. (2022) subjected participants with Lynch syndrome to a randomized double-blind treatment with 30 g resistant starch (RS) (n = 463) daily or placebo (n = 455) for up to 4 years. Upon following up with the participant for up to 20 years, they concluded that 30 g daily RS confers substantial protection against non–colorectal cancers for the group of patients with Lynch syndrome.

So far, five studies have applied personalized medicine strategies in understanding Diabetes and Cardiovascular Diseases by demonstrating variability in treatment response among the patients. In detail, van der Leeuw et al. (2015) attempted to identify which group of patients benefited most from blood pressure-lowering treatment in a large type 2 diabetes mellitus clinical trial. They developed a multivariable treatment model for predicting patients who benefited most from the therapy in terms of absolute risk reduction of major adverse cardiovascular events. Their findings reported the usefulness of using multivariable treatment algorithm in personalized medicine as the algorithm can be used to identify groups of patients that will be subject to blood pressure-lowering therapy. Another study, conducted by Vishram et al. (2015) used 8,505 patients randomized to two kinds of hypertension treatments: losartan and atenolol-based treatment. They tested the potential association between blood pressure (BP) variability and target organ damage. Vishram et al. (2015) concluded that the diastolic BP is associated with stroke. However, there was no statistical evidence for the association between diastolic BP and myocardial infarction. In 2016, Blyme et al. (2016) investigated connections between inflammation and aortic valve stenosis in 1,423 patients. Their findings reported that the increasement of high-sensitivity C-reactive protein (hsCRP) during the first year can be used to predict aortic valve replacement. Thus, the group of patients with increase in hsCRP are more likely to be subject to aortic valve replacement. In 2017, Akintunde et al. (2017) tested the possibility of using physiology-based individualized therapy to treat hypertension. The study sample included patients with uncontrolled hypertension from African countries, including Nigeria, Kenya, and South Africa. Their findings reported that using physiology-based individualized therapy considerably improved blood pressure control among African patients with uncontrolled hypertension. Also, they recommended applying their approach with other African ancestries, i.e., African American, or to all patients with resistant hypertension. In 2019, Owolabi et al. (2019) carried out a randomized stroke trial in Nigeria to determine the effect of a Tailored Hospital-based Risk reduction to Impede Vascular Events after Stroke intervention on blood pressure control among a group of patients with stroke. They concluded that the intervention did not significantly reduce systolic BP among groups of patients with stroke.

Regarding Female sexual interest/arousal disorder, two studies have been done so far and these studies examine the variation in response to treatment among the participating subjects. Using a double-blind, randomized, placebo-controlled crossover study, Tuiten et al. (2018) designed and tested a new genotyping procedure for predicting which drugs will yield a positive treatment response. A total of 139 female sexual interest/arousal disorder patients were treated with three different on-demand drug combinations for three 2-week periods. The study was able to predict which group of women benefits from which on-demand drug. This study could be potentially useful clinically as a companion diagnostic through a personalized medicine approach. In addition, Tuiten et al. (2018b) investigated the efficacy and safety of 2 novel on-demand drugs designed to treat 2 subgroups of patients with female sexual interest/arousal disorder (FSIAD). They used a personalized medicine approach through an allocation formula involving genetic, hormonal, and psychological variables for predicting drug effectiveness in the two subgroups. They reported that drugs under investigation were well tolerated, safe and largely resulted in more satisfying sexual events in the two subgroups.

Regarding Glaucoma disease, Bonnemaijer et al. (2018) conducted a genome-wide association study in 1,113 cases of Primary open-angle glaucoma (POAG) and 1826 controls. They studied samples from Tanzanian, South African, and African American. Their findings reported POAG loci that were significantly associated with POAG in the different groups of the studied samples.

Two studies have demonstrated the application of personalized medicine in HIV disease. The participating samples in these two studies were grouped based on disease status or based on their response to treatment. In detail, Langaee et al. (2021) investigated the relationship between genetic variations in relevant drug disposition genes and Nevirapine (NVP) pharmacokinetics parameters in children living with HIV in Ghana and eligible to receive NVP-based antiretroviral therapy. The subjects were treated with NVP plus zidovudine and lamivudine or abacavir and lamivudine twice daily, TB-coinfected patients got concurrent anti-TB therapy with NVP. Langaee et al. (2021) concluded that genotyping for SNPs encoding the transcriptional factor, pregnane X receptor, improves the prediction of NVP for individualized therapy. In addition, Pujol-Hodge et al. (2022) carried out a universal test-and-treat trial known as the Sustainable East Africa Research in Community Health (SEARCH) trial. They targeted individuals in Uganda and Kenya to reduce regional incidence of HIV-1. They investigated the distribution of HIV-1 drug resistance sequence and HIV-1 subtypes in both groups of population. The study findings emphasized the necessity of improving delivery and adherence to current antiretroviral therapy recommendations, to stop HIV-1 transmission.

Obesity-related disease studies showed how targeting medical interventions can result in changes in health outcomes. Hvidt et al. (2014) investigated the weight changes effects on ambulatory BP in 61 severely obese patients. The patients were subjected to lifestyle intervention and examined with ambulatory BP monitoring at start date and after a year’s treatment. The study concluded that changes in ambulatory BP are linked with different groups of obese patients. Moreover, Hvidt et al. (2015) investigated the impact of weight loss on aortic stiffness in 72 obese patients. However, they reported that the effect of reduction in weight on aortic stiffness was unclear in the different groups of obese patients.

Regarding Parkinson’s disease, Prasuhn et al. (2019) performed a study using a double-blind, randomized, and placebo-controlled approach focused on genetically stratified subgroups of Parkinson’s disease patients (PD) with enrichment of risk variants in mitochondrial genes. This group of patients is more likely to benefit from treatment with the coenzyme Q10, i.e., a mitochondrial enhancer. This study may be an initial step in successfully predicting treatment response on the basis of the genetic status of PD patients thereby translating progress in molecular genetics into personalized patient care.

Regarding Sclerotic disease, du Plooy et al. (2015) studied the independent relationship of fibulin-1 with the inflammatory markers of atherosclerosis. Their study sample involved 290 Africans, i.e., bi-ethnic South African population, and 343 sex- and age-matched Caucasians. This study investigated the utility of personalized medicine based on the underlying ethnic groups of the participants, i.e., participants are grouped based on their ancestry information. Their results indicated that the South African men with lower albumin levels are more likely to have cardiac fibrosis or vascular sclerosis.

## 3 Progress of personalized medicine in Africa

Significant progress has been recorded so far in various aspects related to the implementation of personalized medicine in Africa. Some of these aspects include education system and training, infrastructure, and African-specific data generation. As a result, there has been a recent rise in the number of skilled human resources, infrastructure, and the quality of genomics research emanating from Africa and led by Africans (Uthman et al., 2015).

Since genomics is pivotal to the development of an effective system for personalized medicine (McGonigle, 2016), investments in the training and education of healthcare practitioners among other investments have been recommended as a path to harnessing the opportunities in genomics and precision medicine for the advancement of healthcare (Crellin et al., 2019). Therefore, efforts have been made to provide notable genomics initiatives in Africa. For instance, many organizations such as the Institute of Human Virology, African Collaborative Center for Microbiome and Genomics Research, African Research Group for Oncology, African Center for Translational Genomics, and Center for Genomic and Precision Medicine are among the initiatives taking bold steps in this direction. Refer to Table 2 for examples of prominent genomics initiatives in Africa that are making strong statements in genomics thereby providing frameworks and policies for personalized medicine in Africa. Noting that this list is not intended to be an exhaustive one, but rather a snapshot of a burgeoning list of genomics initiatives in Africa. Although these initiatives and several others have recorded some level of success in advancing the field of genomics in Africa, a lot still remains to be done to fully tap into the wealth of genetic diversity domiciled on the continent towards personalizing medicine for Africans and advancing global health.

**TABLE 2:** showing a few prominent genomics initiatives in Africa.

S/N	Initiative	Description	African countries
**1**	The Human Heredity and Health in Africa (H3Africa) (https://h3africa.org/index.php/about/vision/)	H3Africa seeks to foster human genomic research and improve healthcare in Africa, under the leadership and management of African investigators and their collaborative partners. This initiative has been described as a tipping point for the development of bioinformatics, genomics, and health research in Africa (Adoga et al., 2014)	**North Africa:** Morocco (MAR), Tunisia (TUN), Sudan (SDN), Egypt (EGY)
			**Western Africa**: Nigeria (NGA), Ghana (GHA), Burkina Faso (BFA), Mali (MLI), Gambia (GMB), Ivory Coast, aka côte d’ivoire (CIV), Senegal (SEN), Guinea (GIN), Benin (BEN), Sierra Leone (SLE)
			**Central Africa:** Cameroon (CMR), Democratic Republic of Congo (COD)
			**Eastern Africa:** Ethiopia (ETH), Kenya (KEN), Mauritius (MUS), Tanzania (TZA), Eritrea (ERI), Rwanda (RWA), Madagascar (MGD), Zambia (ZMB), Uganda (UGA), Malawi (MWI)
			**Southern Africa:** South Africa (ZAF), Swaziland, aka Kingdom of Eswatini (SWZ), Zimbabwe (ZWE), Botswana (BWA)
**2**	H3ABioNet The Pan African Bioinformatics Network for H3Africa consortium (H3ABioNet) (https://www.h3abionet.org/about/consortium)	H3ABioNet’s primary objective is to foster bioinformatics expertise in Africa, with a particular focus on facilitating genomics data analysis for H3Africa researchers throughout the continent. The organization is committed to strengthening human capacity by offering comprehensive training and assistance in data analysis. Additionally, H3ABioNet plays a crucial role in enhancing accessibility to informatics infrastructure by creating and providing pipelines and tools for analyzing human, microbiome, and pathogen genomic data	**North Africa:** Morocco (MAR), Tunisia (TUN), Sudan (SDN), Egypt (EGY)
			**Western Africa:** Nigeria (NGA), Ghana (GHA), Senegal (SEN), Mali (MLI)
			**Eastern Africa:** Uganda (UGA), Kenya (KEN) Tanzania (TZA), Malawi (MWI), Mauritius (MUS)
			**Southern Africa:** South Africa (ZAF), Zimbabwe (ZWE), Botswana (BWA)
**3**	The African Centre of Excellence for Genomics of Infectious Diseases (ACEGID) (https://acegid.org/)	ACEGID is a research center in Nigeria focused on genomics and infectious diseases. It conducts advanced research, develops genomic tools and technologies, and provides training and capacity building in genomics and infectious disease research. It also collaborates with local and international partners to address infectious disease challenges in Africa	Nigeria (NGA)
**4**	Medical Research Council (MRC)/Uganda Virus Research Institute (UVRI) and LSHTM Uganda Research Unit (https://www.lshtm.ac.uk/research/units/mrc-uganda)	In 2011, the Medical Research Council (MRC)/Uganda Virus Research Institute (UVRI) and LSHTM Uganda Research Unit initiated the Uganda Genome Resource (UGR) with the aim of promoting the genetic epidemiology of both communicable and non-communicable diseases (NCDs) in Uganda	Uganda (UGA)
**5**	Covenant Applied Informatics and Communication Africa Centre of Excellence (CApIC-ACE) (https://ace.covenantuniversity.edu.ng/)	CApIC-ACE aims to bring genomics research to populations in Africa, customized to address specific issues of health. This is being addressed through enhancing research capacity, improving health education, and increasing treatment efficiency. It also strives to develop new diagnostic biomarkers for prostate and breast cancers as well as drug candidates for malaria	Nigeria (NGA)
**6**	The Nigerian Bioinformatics and Genomics Network (NBGN) (http://www.nbgnetwork.org/)	The Nigerian Bioinformatics and Genomics Network (NBGN) is an initiative dedicated to promoting collaborative research and skill development among bioinformatics and genomics investigators in Nigeria. Its primary goal is to advance and sustain the fields of genomics and bioinformatics within the country. NBGN serves as a platform for fostering collaboration and facilitating interactions between different interdisciplinary subfields, such as genomics, computational biology, and bioinformatics, thereby providing new opportunities for growth and innovation	Nigeria (NGA)
**7**	54Gene (https://54gene.com/)	54gene is a genomic research and health technology company based in Nigeria that focuses on advancing precision medicine in Africa. The company aims to address the challenge of low representation of African populations in genomics research and develop personalized healthcare solutions for diverse populations on the continent. With its innovative approach, 54gene is contributing to the progress of personalized medicine in Africa	Nigeria (NGA)
**8**	Malaria Genomic Epidemiology Network (MalariaGEN) (https://www.malariagen.net/)	Malaria Genomic Epidemiology Network (MalariaGEN) is an international research consortium that aims to understand the genetic factors influencing malaria susceptibility, drug resistance, and transmission. By studying the genomic diversity of malaria parasites and their human hosts, MalariaGEN seeks to improve malaria control and elimination strategies. This collaborative effort plays a crucial role in combating one of the world’s most significant infectious diseases	Africa countries are: Angola (AGO), Benin (BEN), Bioko Island (GNQ), Equatorial Guinea (GNQ), Burkina Faso (BFA), Cameroon (CMR), Central African Republic (CAF), Democratic Republic of the Congo (COD), Ethiopia (ETH), Gabon (GAB), Ghana (GHA), Guinea (GIN), Ivory Coast, aka côte d’ivoire (CIV), Kenya (KEN), Madagascar (MGD), Malawi (MWI), Mali (MLI), Mauritania (MRT), Mayotte Island, aka Grande-Terre (MYT), Mozambique (MOZ), Nigeria (NGA), Senegal, Sierra Leone (SLE), South Africa (ZAF), Sudan (SDN), Tanzania (TZA), Gambia (GMB), Uganda (UGA), Zambia (ZMB), Zimbabwe (ZWE)
**9**	TrypanoGEN. (http://www.trypanogen.net/index.html)	TrypanoGEN is a research initiative focusing on understanding the genetic factors influencing susceptibility to African trypanosomiasis, also known as sleeping sickness. TrypanoGEN aims to develop targeted interventions and improve disease control strategies by studying the parasite as well as the human genomes. This collaborative effort contributes to the fight against a neglected tropical disease affecting vulnerable populations in sub-Saharan Africa	Three hubs
			1. Uganda (UGA) (Makerere University)
			2. Burkina Faso (BFA) (the Centre International de Recherche Developpement sur L’Elevage en Zone Subhumide (CIRDES))
			3. Democratic Republic of the Congo (COD) (Institut National de Recherche Biomedicale (INRB))
**10**	Southern African Human Genome Programme (SAHGP). (https://sahgp.sanbi.ac.za/)	The Southern African Human Genome Programme (SAHGP) is a multidisciplinary effort focused on studying genetic diversity among ethnographic groups in Southern Africa	South African (ZAF)
**11**	Africa Pathogen Genomics Initiative. (https://africacdc.org/thematic-area/africa-cdc-institutes/africa-pathogen-genomics-initiative/)	The Africa CDC, in partnership with the African Society for Laboratory Medicine (ASLM) and regional centers of excellence, implements the Pathogen Genomics and Bioinformatics Fellowship Program. This program enhances the capacity of national public health institutions and laboratories in African Union nations to utilize pathogen genomic data for detecting outbreaks and in disease surveillance	All African Union Member States with 5 Regional Coordination Centres as follows*
			Central Africa: Gabon
			Eastern Africa: Kenya (KEN)
			Northern Africa: N/A
			Southern Africa: Zambia (ZMB)
			Western Africa: Nigeria

● Africa CDC, grouped African Union Member States into the following 5 Regions.

^a^ Central Africa RCC: republic of burundi, Republic of Cameroon, Central African Republic, Republic of Chad, Republic of Congo, Democratic Republic of Congo, Republic of Equatorial Guinea, Gabonese Republic, Democratic Republic of São Tomé and Principe.

^b^ Eastern Africa RCC: union of the comoros, Republic of Djibouti, State of Eritrea, Federal Democratic Republic of Ethiopia, Republic of Kenya, Republic of Madagascar, Republic of Mauritius, Republic of Rwanda, Republic of Seychelles, Federal Republic of Somalia, Republic of the Sudan, Republic of South Sudan, United Republic of Tanzania, Republic of Uganda.

^c^ Northern Africa RCC: People’s Democratic Republic of Algeria, Arab Republic of Egypt, Libya, Islamic Republic of Mauritania, Kingdom of Morocco, Sahrawi Arab Democratic Republic, Republic of Tunisia.

^d^ Southern Africa RCC: republic of angola, Republic of Botswana, Kingdom of eSwatini, Kingdom of Lesotho, Republic of Malawi, Republic of Mozambique, Republic of Namibia, Republic of South Africa, Republic of Zambia, Republic of Zimbabwe.

^e^ Western Africa RCC: republic of benin, Burkina Faso, Republic of Cabo Verde, Republic of Côte d’Ivoire, Republic of Gambia, Republic of Ghana, Republic of Guinea, Republic of Guinea-Bissau, Republic of Liberia, Republic of Mali, Republic of Niger, Federal Republic of Nigeria, Republic of Senegal, Republic of Sierra Leone, Togolese Republic.

## 4 Challenges in the application of personalized medicine in Africa

Although many efforts at implementing personalized medicine in Africa have been addressed, there are still several challenges in this area, which have been summarized below.

### 4.1 Application of genomics in addressing health issues

The challenges associated with the rising incidence of non-communicable diseases, the prevalence of infectious diseases together with the widely known fact of Africa’s wide genetic diversity (Schlebusch et al., 2012), diverse environmental and climatic conditions, dietary and cultural backgrounds (Ibrahim and Bekele, 2019), require that serious focus be given to the research and application of genomics in addressing health issues in clinical settings on the continent. Despite the fact that different kinds of literature have identified the huge prospects associated with harnessing genomics in the African context (Fatumo, 2020), not so much satisfactory work, in comparison with other populations, has been done so far in this regard (Gurdasani et al., 2015; Zhang et al., 2022). This is primarily attributed to the fact that many low- and middle-income countries (LMICs), especially those in Africa, are still in the early stages of development and lack sufficient knowledge and implementation of genomics-based approaches for diagnostic and therapeutic purposes. (Drake et al., 2018).

However, research facilities such as ACEGID at the Redeemer’s University, CApIC-ACE at Covenant University, both in Nigeria, and the Medical Research Council/Uganda Virus Research Institute (MRC/UVRI) and London School of Hygiene and Tropical Medicine (LSHTM) research unit in Uganda, to mention but a few, are blazing the trail in genomics on the continent. ACEGID is particularly interested in the genomics of infectious diseases in Africa. They were instrumental in the first genome sequencing of SARS-COV-2 in Africa (Ihekweazu et al., 2020). CApIC-ACE seeks to develop new diagnostic biomarkers for prostate and breast cancers. They are also building a federated genomics (FEDGEN) infrastructure customized to process and analyze indigenous genomic data (Adetiba, E. et al., 2022). The MRC/UVRI and LSHTM Uganda Research Unit is promoting the genetic epidemiology of both communicable and non-communicable diseases (NCDs) in Uganda. Notwithstanding, more still needs to be done to set Africa in motion for large-scale genomic and personalized medicine adoption.

It is also noteworthy to mention that initiatives such as the H3Africa Consortium are currently doing a lot in bridging the genomics research gap. The consortium has invested in the establishment of high-quality biorepositories in Africa, a bioinformatic network, and a strong training program that has developed skills in genomic data analysis and interpretation among bioinformaticians, wet-lab researchers, and healthcare professionals (Mulder et al., 2018). This initiative aims to enhance genomics capacity and infrastructure across the continent by supporting numerous cutting-edge research projects into type-2 diabetes (T2D), cardiometabolic disease, rheumatic heart disease, chronic kidney disease, neurological diseases, microbiomes, various infectious diseases, and pharmacogenomics, among others—(http://h3africa.org/projects). There is also the Southern African Human Genome Programme (SAHGP) which is an “initiative that aspires to unlock the unique genetic character of southern African populations for a better understanding of human genetic diversity” (Choudhury et al., 2017). The outcomes of all these research endeavors hold significant potential to positively transform genomic medicine, not only within Africa but also on a global scale.

### 4.2 Shortage of skilled personnel

As high-throughput sequencing technologies become more widely accessible, genomic datasets are growing larger and more complex. Thus, there is a pressing need to train a significant mass of African researchers with the requisite skills and expertise to be able to contribute to research in this area rather than just being data generators. Knowledge deficit among healthcare professionals is also a huge barrier to the implementation of personalized genomic medicine. Most clinical practitioners are out-of-touch with the latest trends in genomics technology and many are unable to interpret genetic testing results. Also, several studies (even in developed countries) have shown that a significant number of non-genetics providers and specialists have acknowledged having limited knowledge of genomics and an insufficient capacity to interpret genomic information for their patients. (Bonter et al., 2011). Thus, in the face of the rapidly evolving body of knowledge in this field, it is imperative to recognize that 21st century physicians need more than a fundamental knowledge of human genetics, but a more robust understanding of it (Dhar et al., 2012). Apart from these, insufficient funding for health and education has been largely responsible for patients presenting at health facilities at later stages of diseases with complications that make treatment extremely difficult to manage (Drake et al., 2018).

To effectively utilize next-generation sequencing and other innovative technologies in personalized medicine, it is crucial to have a solid foundation of skills, knowledge, and infrastructure to effectively apply genetic information in healthcare (Ghoorah et al., 2021). Concerted efforts must be put in place in terms of capacity building to create a significant pool of bioinformaticians for biomedical data analysis and provision of genomic medicine training programs for healthcare professionals (Goetz and Schork, 2018). Moreover, emphasis should be placed on more training activities that are participatory in nature such as mentorships, internships, workshops, hackathons, fellowships, and conferences where early career investigators can interact with experts and learn the needed skills and knowledge through practical hands-on applications of concepts bordering around genomics and personalized medicine.

### 4.3 Infrastructure

The lack of adequate infrastructures and technologies to support research and clinical translation has been identified as another major barrier to the adoption of personalized genomic medicine in Africa (Mulder, 2017; Jongeneel et al., 2022). To facilitate comprehensive DNA analysis, Africa should prioritize improving access to technology for researchers. Investment is required in various areas to support DNA sequencing and genotyping facilities, establish biobanks for sample storage along with their associated data, and develop robust data infrastructure and information management systems for efficient data generation, storage, and analysis pipelines. Additionally, there is a need to improve electronic health records, ensure reliable internet connectivity, and establish facilities for clinical action and conducting clinical trials.

### 4.4 Research

Genomic medicine aims to reveal an individual’s genetic predisposition to diseases, usually by establishing a link between genotype and phenotype through genome-wide association studies (GWAS). Although prediction methods such as polygenic risk scores (PRS) hold potential for clinical applications, individuals of non-European descent may not benefit much from genomic medicine due to their current underrepresentation in extensive genetics and genomics research (Ju et al., 2022). For genomic medicine to translate into personalized care globally, as many different populations as feasible must be sufficiently represented in genomic research (Bentley et al., 2019). It has been suggested that failure to prioritize genomic studies in Africa, which harbors the highest genetic diversity among human populations, would hinder global health equity. Thus, there is a need for more data on African population genomics to address this challenge. Also, it is expedient for extensive research to be done to develop databases and other platforms for easy access to data on genomic variants and their associated genes and diseases (Bombard, 2015), especially among African populations.

Most genomic research involves the generation as well as the analysis of genomic data which can be quite expensive in terms of cost, time, and computational resources required. Most of these costs can be offset if there are huge investments in place for funding research work in these areas. Most African genomic scientists and researchers operate in relatively resource-scarce environments and largely lack the needed capacity to contribute and compete effectively with their counterparts in larger and better-resourced groups in the analysis of genomics data. Therefore, many of them resort to data generation in their quest to at least make any contribution. Thus, adequate funding will go a long way to address this challenge.

It is very crucial for deliberate efforts to be made at developing genomics research capacity in Africa. For instance, there is a serious need for collaborations among African investigators as well as with researchers in high-income settings who have the capacities, expertise, and funding to drive research. Mechanisms can also be put in place to allow government authorities, industrial partners, and/or researchers in different African countries to pool resources together in establishing and strengthening regional research centers and research networks with common interests instead of working in isolation (Tekola-Ayele and Rotimi, 2015).

### 4.5 Data generation and data sharing

Populations with non-European ancestry have not kept pace with their European ancestry counterparts, partly due to insufficient representation as both research participants and researchers in genomics research (Fatumo, 2020). More research and data that is applicable to indigenous populations in Africa is needed. This could help prevent the mistaken consideration of African individuals for disease risk variants that seem to be overrepresented in African populations (Mulder, 2017). African investigators need to be equipped to play vital roles in the global arena of genomic research so that Africa is not left out in the current waves of personalized precision medicine. Also, more public enlightenment must be done to encourage more members of the public to volunteer as subjects in genetic studies. Ethical and societal issues surrounding the use of genomic technologies should also be properly addressed through adequate education, orientation, and legislation where necessary.

### 4.6 African diversity and the African reference genome

The central importance of Africa to the origin of man cannot be overemphasized. The continent, which is the second largest in the world in terms of size and population, accommodates diverse ethnicities. For instance, Nigeria, which is the most populous and most diverse country in Africa and has 250 ethnic groups with over 500 different native languages, is characterized by high levels of genetic diversity and a broad population substructure (Fatumo et al., 2020). A recent study by Joshi et al. (2023) described the whole-genome sequencing of 449 Nigerian individuals across 47 unique self-reported ethnolinguistic groups in the country. Their results emphasize the value of the African genomic resource in improving our understanding of human ancestry and health.

As a matter of fact, obtaining an accurate representation of the reference genome is the cornerstone of the personalized medicine revolution. Thus, there have been discussions in the scientific community on the need for a more representative reference genome, especially in populations with wide genetic diversity. This is with the hope that such will allow for more utility and compatibility of the outcomes of whole genome sequencing in such populations and reduce biases. For instance, results from the 1,000 Genomes dataset reveal that the African super-population genomic data has the largest divergence from the GRCh38 reference (Tetikol et al., 2022). In fact, Sherman et al. (2019) also observed that the African pan-genome used in their study contains ∼10% more DNA than the current human reference genome. Therefore, different methods have been proposed to address this disparity in the reference genome and the population genomic data during large sequencing projects. Some of these approaches include: adding and extending nucleotides in the existing reference (Huang et al., 2013); generating a population-specific consensus sequence by assembling raw read data from scratch (Duan et al., 2019; Sherman et al., 2019), and graph-based references that have the ability to simultaneously represent multiple distinct populations. Tetikol et al. (2022) proposed a population-specific graph construction method to ensure that a more representative reference is used for the downstream analysis of large-scale genome sequencing projects. Since graph genomes offer more genetic diversity, they provide a viable framework for assembling African-specific references that would significantly reduce the biases of the GRCh38 reference.

### 4.7 Health systems restructuring

In many healthcare settings in Africa, doctors and other healthcare providers still depend largely on the “one-size-fits-all” generalized approach of administering care rather than considering the individual uniqueness of each patient in care decisions. Also, most health facilities are poorly funded and understaffed. As a result, they lack the infrastructure and capacity for individualized healthcare delivery. Thus, it is important to adequately reimagine and reconfigure the health system on the continent to meet up with global best practices.

For personalized medicine to be successful, it requires sufficient infrastructure within the healthcare system to support the research and implementation of targeted diagnosis and treatment processes. For instance, a couple of health facilities across the world are using sequencing to unravel maladies that have defied diagnosis, including life-threatening conditions, particularly among children and the elderly (Worthey et al., 2011; Manolio et al., 2013; Gorzynski et al., 2022). Hence, it is necessary that national and sub-national frameworks are put in place for the adoption of routine sequencing, electronic medical records (EMR), and other standard practices to effectively improve the health outcomes of African communities. Health systems should also be ready to embrace research as there is also the possibility of key actors in the health sector failing to adopt research-proven interventions (Goetz and Schork, 2018). Resistance to change rather than lack of information or resources sometimes could be the problem impeding the deployment of certain interventions. Therefore, there must be willingness and openness to adopt current trends in clinical care.

## 5 Conclusion

Personalized medicine, if well implemented, holds the promise of being more cost-effective than the conventional traditional medical practices. It also has the potential of improving the quality of life of the patients. Diversity in genomics research is vital for both social justice and scientific advancement. Because of its rich genetic diversity, Africa needs to be well fortified with the right resources and technologies to be able to combat most of its health issues. However, this responsibility of adapting personalized medicine into the healthcare system in Africa should not be seen only as an “African issue to address”. It is a global endeavor that necessitates partnerships with diverse communities throughout the research process, dissemination of findings, and utilization of new technologies. Therefore, international partners (in the form of funding organizations, research collaborators, etc.), as well as individuals with African ancestry, have diverse but important roles to play in this global venture, as already highlighted in this review. Thus, all hands must be on deck to ensure its success in the African context.

Based on the reviewed scientific literature, we would like to share the following considerations to successfully apply personalized medicine concepts on a pan-African level. First, to enable personalized genomic medicine to flourish in Africa, it is essential to have inclusive discussions among stakeholders from clinical, research, industry, non-governmental, and legislative sectors. These dialogues should result in the development of comprehensive national, regional, and continental policies, as well as research infrastructure. These policies and infrastructure are crucial for advancing research, improving patient care, and ensuring fair access to new genomic technologies. The integration of genomics into the educational and health system of African communities is pivotal to the actualization of individualized medicine as it will create the human capital that will drive development in this field. Second, the implementation of personalized medicine would require the capacity building of a diverse array of skilled personnel. These include researchers, doctors, nurses, clinical geneticists, genetic counselors, pharmacists, bioinformaticians, biostatisticians, technicians, data scientists, and data security personnel. Collaborative and synergistic efforts must be made to harness the expertise of these personnel to develop personalized medicine on the continent. Also, the skilled diaspora community should be deliberately attracted to tap into their expertise by providing an enabling environment and investment in infrastructure. Third, the stakeholders, including health authorities, need to provide clear policies for personalized medicine applications to guide research in their research lines.
